# Development International Classification of Functioning, Disability and Health Core Set for Post Total Knee Replacement Rehabilitation Program: Delphi-Based Consensus Study in Taiwan

**DOI:** 10.3390/ijerph18041630

**Published:** 2021-02-09

**Authors:** Shih-Wei Huang, Yi-Wen Chen, Reuben Escorpizo, Chun-De Liao, Tsan-Hon Liou

**Affiliations:** 1 Department of Physical Medicine and Rehabilitation, Shuang Ho Hospital, Taipei Medical University, Taipei 23561, Taiwan; 13001@s.tmu.edu.tw (S.-W.H.); 17304@s.tmu.edu.tw (Y.-W.C.); 08415@s.tmu.edu.tw (C.-D.L.); 2 Department of Physical Medicine and Rehabilitation, School of Medicine, College of Medicine, Taipei Medical University, Taipei 11031, Taiwan; 3 Department of Rehabilitation and Movement Science, College of Nursing and Health Sciences, University of Vermont, Burlington VT 05401, USA; escorpizo.reuben@gmail.com; 4 Swiss Paraplegic Research, 6207 Nottwil, Switzerland; 5 Master Program in Long-Term Care & School of Gerontology Health Management, College of Nursing, Taipei Medical University, 250 Wu-Xing Street, Taipei 11031, Taiwan; 6 Department of Physical Medicine and Rehabilitation, Wan Fang Hospital, Taipei Medical University, Taipei 11600, Taiwan

**Keywords:** total knee arthroplasty (TKA), rehabilitation, International Classification of Functioning, Disability and Health, core set

## Abstract

Osteoarthritis is one of the leading causes of disability. Total knee arthroplasty (TKA) is a surgical intervention for patients with severe osteoarthritis. Post TKA rehabilitation is crucial for improving patient’s quality of life. However, traditional rehabilitation has only focused on physical function; a systemic analysis of other dimensions such as social participation and environmental factors of post TKA rehabilitation is lacking. The aim of this study was to develop a core set from the International Classification of Functioning, Disability and Health (ICF) to create a comprehensive rehabilitation program for patients with osteoarthritis post TKA. Before the Delphi-based consensus process, a literature review process was performed for related ICF categories selection. We used a three-round Delphi-based consensus among 20 physical therapists with orthopedic rehabilitation expertise in a university-based hospital. A five-point Likert scale was used to rate the importance of each item. The consensus of ratings was analyzed using Spearman’s rho and semi-interquartile range indices. The ICF core set for post TKA rehabilitation was determined based on a high level of consensus and a mean score of ≥4.0 in the third Delphi-based consensus round. The ICF core set comprised 32 categories, with 13 regarding body function, four regarding body structures, nine regarding activities and participation, four regarding environmental factors, and two regarding personal factors. Our ICF core set for post TKA rehabilitation can provide information on effective rehabilitation strategies and goal setting for patients post TKA. However, further validation and feasibility assessments are warranted.

## 1. Introduction

Osteoarthritis (OA) is a leading cause of walking and stair-climbing limitations among older adults, as well as of disability worldwide [1,2]. Patients with OA present symptoms of pain and stiffness, which affect life quality and limits outdoor activities and social interactions [3]. The OA-related burden on the health system is increasing because of the aging of the population [4]. Total knee arthroplasty (TKA) is a common surgical intervention procedure for end-stage patients with OA with symptoms that are refractory to conservative treatment or present a significant decrement of functions of daily living. The American Joint Replacement registry reports that over 500,000 TKA procedures were performed in the United States in 2016 [5]. The annual incidence of TKA is projected to be 3.5 million in 2030 [6]. Moreover, the international survey of TKA performance revealed a marked increase globally [7].

Despite significant advancements in surgical and prosthesis placement techniques, numerous patients present a limited range of motion (ROM), pain, limited physical function restoration and impaired quality of life [8]. Effective post TKA rehabilitation is crucial in clinical practice for the restoration of function and holistic assessment. To evaluate the limitation of patients with OA, the Western Ontario and McMaster Universities Osteoarthritis Index (WOMAC) and Knee Injury and Osteoarthritis Outcome Score are usually used [9,10]. These scales cannot provide information about social participation and quality of life. To assess quality of life, the Short-Form 36 (SF-36) is frequently used among patients with OA, with convincing validity [11]. Although the SF-36 covers four domains of physical health and four domains of mental condition, it mostly focuses on body functions and provides limited information regarding leisure activities and social participation [12]. Improvements in quality of life and daily leisure activities are also critical among patients post TKA. The World Health Organization provided the International Classification of Functioning, Disability and Health (ICF) framework for a more comprehensive and holistic description of the functioning and disability status of patients [13]. The ICF contains body function, body structure, activity and participation components. These components reflect patients’ health conditions, personal interactions, and social interactions.

The ICF provides disease classification and a systemic approach to identify health-related problems and conditions. However, it includes over 1450 categories, which limits its clinical application [14]. More precise ICF categories describing the disability status of specific patient types are required for clinical application [15]. Rehabilitation programs for patients post TKA frequently focus on physical function. However, quality of life and return to leisure activities and social participation are also critical in reaching post TKA rehabilitation goals. To our knowledge, no brief ICF core set has been developed for post TKA rehabilitation program goal setting and strategy development. Besides, under our health care system, physical therapists performed the post TKA program and are most familiar with which portion of this program should be mentioned. We hypothesized that this brief ICF core set can present the real needs of post TKA patients. Therefore, in this Delphi-based consensus study, we developed a brief ICF core set by physical therapists to reveal the portion that is valuable for effective rehabilitation program setting among patients post TKA.

## 2. Methods

### 2.1. Study Design and Selection of ICF Categories

The Delphi-based consensus method was used to determine the core ICF categories relevant to rehabilitation among patients with TKR [16]. All possible factors related to post TKR rehabilitation were listed following a systematic review and discussion with health care experts regarding rehabilitation after TKR. The post TKR rehabilitation-related factors were then linked to the ICF categories. Finally, three rounds of questionnaires regarding these categories were administered to 20 physical therapy professionals to confirm the categories most relevant to patients post TKR. This study was approved by the Joint Institutional Review Board of Taipei Medical University (N201803052).

Articles concerning rehabilitation and TKR were searched using the keywords “osteoarthritis”, “rehabilitation”, and “knee replacement”. All relevant articles in English were included for further evaluation, and quality assessment was performed. The selected articles were then reviewed independently by two reviewers (Huang and Liao), who selected the TKR rehabilitation-related factors. In case of conflict of opinion among the reviewers, a third reviewer (Liou) judged and determined whether the factors should be included. All relevant factors were then defined and linked to TKR rehabilitation categories based on systematic and standardized procedures. Forty-seven factors related to TKR rehabilitation were linked with ICF categories, with 14, 4, 17, and 12 categories belonging to the codes for body function (b), body structure (s), activities and participation (d), and environmental factors (e), respectively [17]. Two personal factors, age and sex, were also considered in this Delphi consensus process.

### 2.2. Consensus Procedure

**Figure 1:**
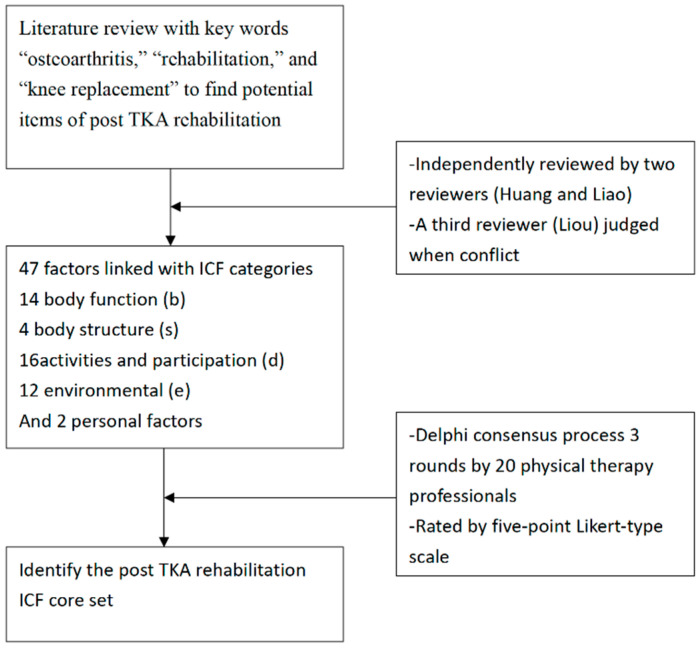
Study flowchart.

The ICF categories related to post TKR rehabilitation were evaluated over three formal rounds of Delphi-based consensus surveys. From 1 August 2019 to 31 December 2019, Delphi-based consensus surveys were administered to 20 physical therapists with expertise in the orthopedic and sports rehabilitation fields. The therapists were invited to participate through emails containing information on the study aim and consensus methods. The questionnaire was mailed to those who agreed to participate. The first questionnaire contained the second-level ICF codes for potential TKR rehabilitation-related categories and detailed descriptions of these categories. The participants were asked to rate the importance of selected categories in post TKR rehabilitation programs on a five-point Likert-type scale (5, very important; 4, important; 3, marginally important; 2, least important; 1, not important). The results of each item in the first round were considered reference data for the second round, including the participants’ scores, all participants’ mean scores and the participants’ standard deviations (SDs). Based on these results, all participants were requested to reevaluate their previous ratings, all participants’ mean scores, SDs, and rate each item again in the second round. Similarly, the results of each item in the second round were considered reference data when answering the five-point Likert-type scale of each item for the third round. The consensus formation was complete upon completion of the three rounds of the Delphi-based consensus method (Figure 1).

### 2.3. Data Analysis

To identify appropriate brief ICF core set categories for patients post TKR, we performed serial data analysis. Analysis of the Spearman’s rank correlation coefficient (rho) was performed to compare participants’ scores with the mean scores of all participants for each ICF category over the three rounds of the Delphi-based consensus process. A Spearman’s rho of >0.7 indicated strong agreement between individual participants and all participants of each panel; *p* < 0.05 was considered statistically significant. The dispersion of each participant’s score was quantified using the semi-interquartile range (SIQR) by dividing between the third and the first quartiles (75th and 25th percentile, respectively). SIQRs were calculated for each item; SIQR ≤ 0.5 indicated a high level of agreement. The importance of each item was based on the mean Likert score; categories with a mean score of ≥4.0 in the third round of the Delphi-based consensus were considered the ICF core set. Data analyses were performed using SPSS (version 17.0; IBM, Armonk, NY, USA).

## 3. Results

**Figure 2:**
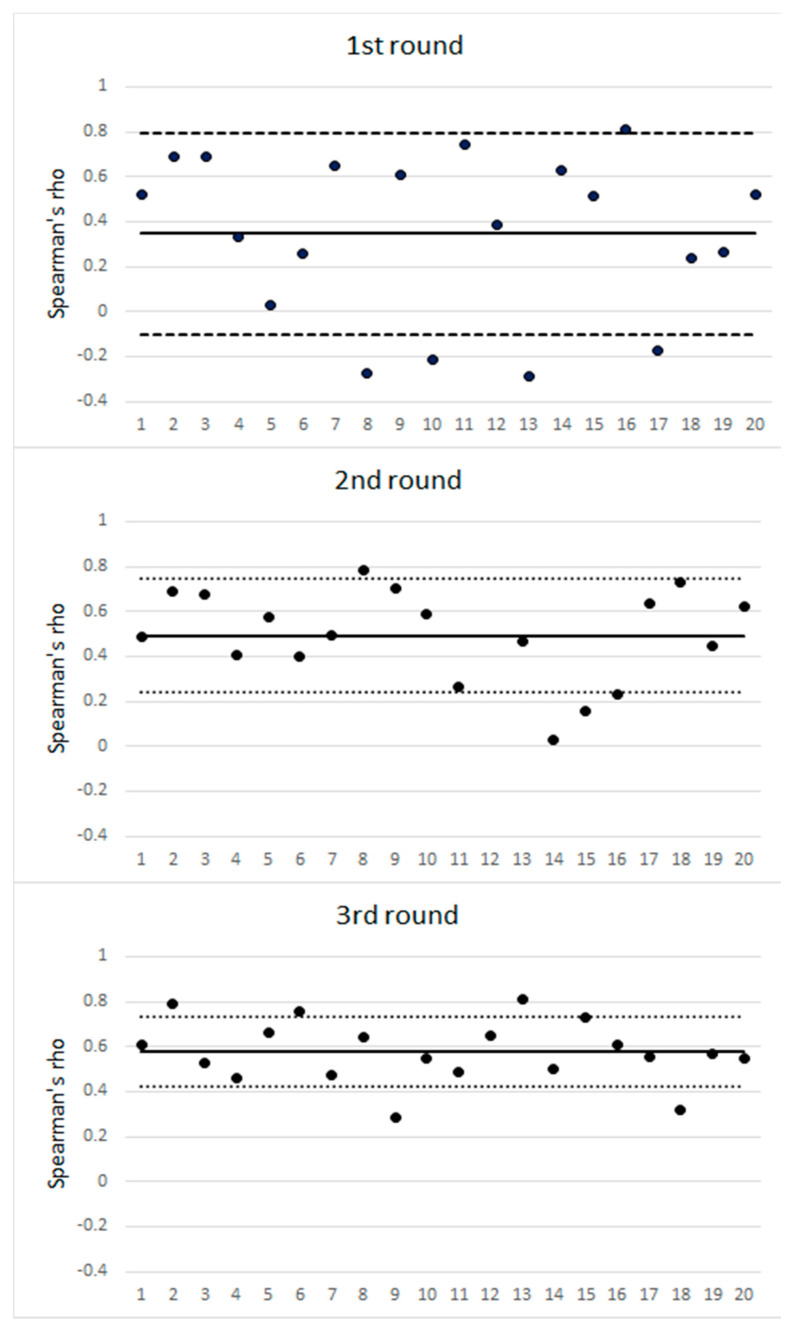
Spearman’s rho for individual expert scores and the team mean score of all responders over three rounds of Delphi-based consensus. The horizontal solid line indicates the mean Spearman’s rho for each round. The area between the dotted lines indicates the mean ± 1 SD.

We recruited 20 physical therapists (11 women and nine men), with expert experience of over five years. All participants completed the three rounds of the Delphi-based consensus. Figure 2 illustrates the Spearman’s rho corresponding to each participant’s scores and the mean scores of all participants in the three rounds. The mean (SD) Spearman’s rho was 0.34 (0.45), 0.49 (0.25), and 0.56 (0.15) in the first, second and third rounds, respectively.

Table 1 presents the results of the third round. A total of 32 categories received scores of >4.0 on the Likert-type scale; these categories were included in the final ICF core set: 13, four, nine, four and two categories belonged to body functions, structure, activities and participation, environmental factors, and personal factors, respectively. The categories of consciousness functions (b110), undertaking and single task (d210), and carrying out daily routine (d230) achieved the highest level of expert consensus (>4.8 points on the Likert-type scale).

Percentage of agreement and disagreement of importance of ICF core set for post TKA rehabilitation is added in Appendix A.

## 4. Discussion

**Figure 3:**
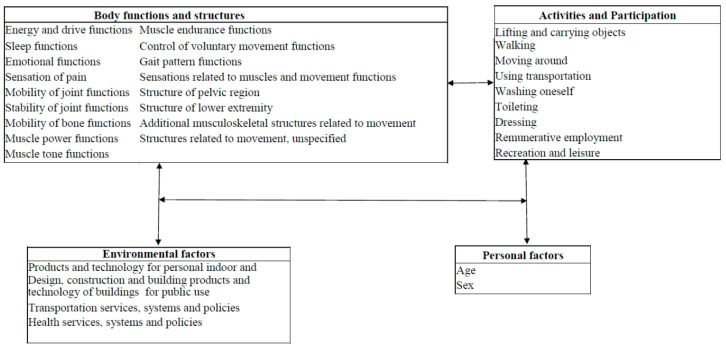
ICF core set of factors associated with post TKA rehabilitations. The arrows presented the association of different categories based on ICF framework.

The WHO provided the ICF framework in 2001. Although the ICF provides disease classification and a systemic approach to identify the health-related problems and conditions, it includes more than 1450 categories, hindering effective clinical application [14]. More precise ICF categories describing the disability status of specific patient types are needed [15]. Therefore, ICF core sets based on specific diseases and health care facility settings can be developed through expert consensus [15,16]. We identified 32 categories of the ICF core set for post TKA rehabilitation using the Delphi consensus process. An effective post operation rehabilitation program is essential to reduce admission days and the delay in returning to daily activities or work among patients receiving TKA. Most studies on TKA rehabilitation programs have focused on regaining physical function after an operation, whereas our study provided an ICF-based core set for rehabilitation strategy setting. Through a Delphi-based consensus process and use of the framework of the ICF, we identified the relevant ICF factors that demonstrated an interaction between environmental factors, physical functioning, participation and post TKA rehabilitation (Figure 3). The concept of ICF is compatible with studies that have reported that multidisciplinary team intervention is more effective among patients post TKA [18]. However, we propose that the strategy for recruiting the intervention team from different expertized fields is also critical. The ICF core set could provide information regarding the subspecialties or professions that should be combined for comprehensive post TKA rehabilitation interventions.

Despite the post TKA pain relief, patients may continue to present muscle atrophy and ROM limitation, which limit daily living activities such as walking, climbing stairs, squatting and exercise [19]. Traditional rehabilitation for patients receiving TKA focuses on regaining ROM and strength, which were involved in physical functions [20]. The core set for patients receiving TKA principally involved physical function categories, which was compatible with our findings. We identified 13 categories of physical function as the core set. In addition to joint-movement–related categories, the perception of pain (b280), the sensation of related muscle and movement functions (b780), energy and drive functions (b130) and emotional and sleep functions (b134, b152) were included in the core set. The categories of the sensation of pain (b280), mobility of joint functions (b720), stability of joint functions (b715) and muscle power functions (b730) were above 4.5 on average on the five-point Likert scale. The physical function categories remained essential for post TKA rehabilitation. However, the energy, sleep and emotional aspects also warranted concern when treating these patients. Psychological support and sleep quality support are also crucial aspects of multidisciplinary rehabilitation interventions. This support can be offered by providers with other specialties. A study reported that sleep quality declined during the first six weeks post TKA, which was compatible with our core set [21].

In the aspect of body structures, motion-related structures other than the knee joint, such as the pelvic region, are also critical for post TKA rehabilitation. A study mentioned that trunk control can be improved post TKA [22]. However, another study reported that some patients receiving TKA retained spinal pelvic imbalance after surgery [23]. Therefore, the pelvic portion is central to restoring function and should thus be included in a post TKA rehabilitation program.

The ICF core set included nine categories associated with activities and participation. These categories were as follows: carrying objects (d430), walking (d450), moving around (455), washing oneself (d510), toileting (d530), dressing (d540), participating in remunerative employment (d850) and participating in recreation and leisure (d920). Daily activities and participation are paramount in enabling patients receiving TKA to return to their previous lifestyle and in improving their life quality. Studies have reported that TKA care generally improves the quality of life and leads to favorable functional outcomes [24,25]. However, another study reported that most existing research has focused on functional aspects or activity levels and lacks broader constructs regarding the patient quality of life [26]. Our ICF core set provided other daily activity aspects that should be considered in post TKA rehabilitation. Dressing, toileting, washing, participating in employment and participating in leisure activities were determined to be crucial in rehabilitation program goal-setting. Occupational therapists could be enrolled for post TKA multidisciplinary team intervention.

Our TKA rehabilitation core set contained four environment aspect categories, which were as follows: products and technology for personal indoor and outdoor mobility and transportation (e120); design, construction, and building products and technology of buildings for public use (e150); transportation services, systems, and policies (e540) and health services, systems, and policies (e580). We determined that health service and health policy systems were included in the core set in addition to transportation and building technology. Rehabilitation goal-setting can be related to the use of transportation and a safe environment for daily living. Furthermore, health policy support systems were associated with effective rehabilitation post TKA. Transportation and building technologies were related to a higher quality of life after TKA rehabilitation. However, public health medical resources are crucial for post TKA rehabilitation programs. Environmental factors should also be considered to regain function after surgery and thus obtain effective and efficient rehabilitation post TKA.

Compared with previous studies about ICF comprehensive core sets with 55 categories for osteoarthritis, which contained 13 categories from the component body functions, six from the component body structures, 19 from the component activities and participation and 17 from the component environmental factors, we presented more concise ICF core sets with 32 categories [27]. Comparing the body functions categories, our study is compatible with the same categories of both core sets. It indicates that for physical therapists, the categories of body functions of post TKA rehabilitation are similar to osteoarthritis. In the aspect of body structures, there were two categories that mentioned upper extremities (s720 and s730) not related to our study. The most different part of osteoarthritis and our post TKA rehabilitation core sets were activities and participation and environmental categories. Our study focused on post TKA rehabilitation, and we supposed that different circumstances, cultures and environments could lead to the variance of the component of the core set (Table 2).

This study reported an ICF core set that was specifically designed for the post TKA rehabilitation program. However, several limitations must be addressed. First, the perception of the importance of post TKA rehabilitation factors could differ among participants of this study. The different circumstances post TKA and rehabilitation intervention periods could cause perceptional variations in the importance of certain ICF categories. To avoid this discrepancy, the situations of patients with TKA were described before answering the questionnaire. Second, participants in the ICF core set were physical therapists, and TKA may be related to various fields of professionals. However, post TKA rehabilitation is mostly performed by physical therapists in our country; we supposed that makes them more familiar with post TKA rehabilitation. Further studies of Delphi process by other fields of professionals are recommended in the future. Third, the feasibility and validity of the ICF core set were not obtained from patients post TKA. The feasibility and validity of patients post TKA under rehabilitation should be investigated to identify clinical applications in the further study. Finally, the participating experts were limited to Taiwan. The opinions of post TKA rehabilitation programs may vary across countries. Nevertheless, we believe that our core set is applicable to patients not only in Taiwan but also those in other countries with similar medical system. However, further study is needed to investigate the feasibility of this core set for those countries with different health care system and environments.

## 5. Conclusions

Effective post TKA rehabilitation is related to social participation and environmental factors, as well as physical functions. The ICF, a systematic framework, reveals interactions between various dimensions of the rehabilitation of TKA patients. Our Delphi consensus-based ICF core set for patients with TKA can provide information on effective rehabilitation strategies, multidisciplinary team intervention and goal-setting for post TKA rehabilitation programs. Moreover, it could be helpful to develop individualized telerehabilitation program with weight-bearing biofeedback, neuromuscular electrical stimulation and balance control system for outpatient therapy, with personalized intensity and intervention by related professionals under the framework of this core set.

## Figures and Tables

**Table 1:** International Classification of Functioning, Disability and Health (ICF) categories in the ICF core set fort knee arthroplasty (TKA) rehabilitation.

ICF Code	ICF Category Title	Round 1	Round 2	Round 3
Body functions				
b130	Energy and drive functions	3.7 (1.3)	4.3 (0.7)	4.1 (0.5)
b134	Sleep functions	3.6 (1.3)	4.0 (1.0)	4.1 (0.6)
b152	Emotional functions	3.5 (1.3)	4.1 (0.8)	4.0 (0.8)
b280	Sensation of pain	4.0 (1.3)	4.6 (0.6)	4.5 (0.6)
b710	Mobility of joint functions	4.0 (1.4)	4.7 (0.6)	4.8 (0.4)
b715	Stability of joint functions	4.0 (1.3)	4.6 (0.5)	4.6 (0.5)
b720	Mobility of bone functions	3.7 (1.3)	4.5 (0.8)	4.2 (0.8)
b730	Muscle power functions	4.0 (1.4)	4.7 (0.5)	4.6 (0.5)
b735	Muscle tone functions	3.6 (1.1)	4.0 (1.1)	4.0 (0.9)
b740	Muscle endurance functions	3.8 (1.4)	4.5 (0.7)	4.4 (0.7)
b760	Control of voluntary movement functions	3.7 (1.3)	4.5 (0.6)	4.2 (0.8)
b765	Involuntary movement functions	2.9 (0.9)	3.6 (0.6)	3.8 (1.0)
b770	Gait pattern functions	3.6 (1.4)	4.6 (0.5)	4.3 (0.7)
b780	Sensations related to muscles and movement functions	3.8 (1.2)	4.5 (0.5)	4.4 (0.6)
s740	Structure of pelvic region	3.4 (1.2)	4.4 (0.6)	4.1 (0.8)
s750	Structure of lower extremity	3.7 (1.3)	4.5 (0.6)	4.4 (0.6)
s770	Additional musculoskeletal structures related to movement	3.7 (1.4)	4.5 (0.6)	4.1 (0.6)
s799	Structures related to movement, unspecified	3.7 (1.3)	4.5 (0.6)	4.2 (0.6)
Activities and participation				
d430	Lifting and carrying objects	3.8 (1.0)	4.3 (1.0)	4.1 (0.9)
d440	Fine hand use	2.9 (1.2)	3.4 (1.2)	3.2 (1.1)
d445	Hand and arm use	3.1 (1.1)	3.5 (1.1)	3.2 (1.0)
d450	Walking	4.1 (1.4)	4.7 (0.5)	4.7 (0.5)
d455	Moving around	3.6 (1.5)	4.6 (0.6)	4.4 (0.8)
d470	Using transportation	3.9 (1.3)	4.4 (0.7)	4.0 (0.8)
d475	Driving	3.7 (1.0)	4.1 (0.9)	3.5 (0.8)
d510	Washing oneself	3.7 (1.2)	4.4 (0.7)	4.0 (0.7)
d530	Toileting	3.8 (1.3)	4.3 (0.9)	4.2 (0.7)
d540	Dressing	3.8 (1.2)	4.1 (1.1)	4.0 (0.7)
d620	Acquisition of goods and services	3.3 (0.9)	4.0 (0.9)	3.3 (0.6)
d640	Doing housework	3.7 (1.0)	4.1 (1.0)	3.7 (0.7)
d660	Assisting others	3.3 (0.7)	3.9 (0.9)	3.5 (0.7)
d770	Intimate relationships	3.1 (0.9)	3.8 (0.9)	3.4 (0.7)
d850	Remunerative employment	3.8 (1.2)	4.5 (0.7)	4.2 (0.5)
d910	Community life	3.5 (0.9)	4.1 (0.9)	3.8 (0.6)
d920	Recreation and leisure	3.8 (0.9)	4.3 (0.8)	4.1 (0.6)
Environmental factors				
e120	Products and technology for personal indoor and outdoor mobility and transportation	3.9 (1.0)	4.2 (0.7)	4.3 (0.6)
e135	Products and technology for employment	3.6 (0.8)	3.8 (0.7)	3.9 (0.7)
e150	Design, construction and building products and technology of buildings for public use	3.4 (0.8)	3.8 (0.9)	4.0 (0.6)
e155	Design, construction and building products and technology of buildings for private use	3.6 (0.7)	3.8 (0.8)	3.7 (0.6)
e225	Climate	3.0 (0.7)	3.3 (0.9)	3.2 (1.0)
e310	Immediate family	3.2 (1.2)	3.7 (1.0)	3.5 (0.9)
e320	Friends	3.3 (1.0)	3.7 (1.0)	3.6 (0.8)
e450	Individual attitudes of health professionals	3.2 (1.2)	3.6 (1.0)	3.5 (1.0)
e460	Societal attitudes	3.5 (1.1)	3.7 (1.0)	3.8 (0.8)
e540	Transportation services, systems, and policies	3.6 (1.1)	4.0 (0.9)	4.0 (0.8)
e575	General social support services, systems, and policies	3.7 (0.7)	4.0 (0.8)	3.9 (0.7)
e580	Health services, systems, and policies	4.0 (0.8)	4.2 (0.6)	4.2 (0.7)
Personal factors				
	Age	4.6 (0.6)	4.7 (0.6)	4.8 (0.4)
	Sex	4.2 (1.0)	4.1 (0.7)	4.1 (0.6)

Values are 20 experts’ mean (standard deviation) scores on a 5-point Likert-type scale.

**Table 2:** Comparing categories of ICF core set for osteoarthritis and post TKA rehabilitation.

ICF Domain	ICF Core Set for OA	ICF Core Set for Post TKA Replacement
Body functions and structure		
b130 Energy and drive functions	x	x
b134 Sleep functions	x	x
b152 Emotional functions	x	x
b280 Sensation of pain	x	x
b710 Mobility of joint functions	x	x
b715 Stability of joint functions	x	x
b720 Mobility of bone functions	x	x
b730 Muscle power functions	x	x
b735 Muscle tone functions	x	x
b740 Muscle endurance functions	x	x
b760 Control of voluntary movement functions	x	x
b770 Gait pattern functions	x	x
b780 Sensations related to muscles and movement functions	x	x
s720 Structure of shoulder region	x	
s730 Structure of upper extremity	x	
s740 Structure of pelvic region	x	x
s750 Structure of lower extremity	x	x
s770 Additional musculoskeletal structures related to movement	x	x
s799 Structures related to movement, unspecified	x	x
Activity and Participation		
d410 Changing basic body position	x	
d415 Maintaining a body position	x	
d430 Lifting and carrying objects	x	x
d440 Fine hand use	x	
d445 Hand and arm use	x	
d450 Walking	x	x
d455 Moving around	x	x
d470 Using transportation	x	x
d475 Driving	x	
d510 Washing oneself	x	x
d530 Toileting	x	x
d540 Dressing	x	x
d620 Acquisition of goods and services	x	
d640 Doing housework	x	
d660 Assisting others	x	
d770 Intimate relationships	x	
d850 Remunerative employment	x	x
d910 Community Life	x	
d920 Recreation and leisure	x	x
Environmental factor		
e110 Products or substances for personal consumption	x	
e115 Products and technology for personal use in daily living	x	
e120 Products and technology for personal indoor and outdoor mobility and transportation	x	x
e135 Products and technology for employment	x	
e150 Design, construction and building products and technology of buildings for public use	x	x
e155 Design, construction and building products and technology of buildings for private use	x	
e225 Climate	x	
e310 Immediate family	x	
e320 Friends	x	
e340 Personal care providers and personal assistants	x	
e355 Health professionals	x	
e410 Individual attitudes of immediate family members	x	
e450 Individual attitudes of health professionals	x	
e460 Societal attitudes	x	
e540 Transportation services, systems and policies	x	x
e575 General social support services, systems and policies	x	
e580 Health services, systems and policies	x	x

## Data Availability

Data available on request due to restrictions eg privacy or ethical.

## Appendix A

**Table A1:** Agreement and disagreement of ICF categories in the ICF core set for CVA.

ICF Code	ICF Category Title	Round 1	Round 2	Round 3
Body functions				
b130	Energy and drive functions	70 (25)	90 (0)	90 (0)
b134	Sleep functions	60 (20)	75 (5)	90 (0)
b152	Emotional functions	60 (30)	70 (0)	70 (0)
b280	Sensation of pain	70 (20)	95 (0)	95 (0)
b710	Mobility of joint functions	65 (20)	95 (0)	100 (0)
b715	Stability of joint functions	65 (15)	100 (0)	100 (0)
b720	Mobility of bone functions	65 (25)	95 (5)	75 (0)
b730	Muscle power functions	70 (20)	100 (0)	100 (0)
b735	Muscle tone functions	55 (15)	65 (10)	65 (5)
b740	Muscle endurance functions	65 (20)	90 (0)	90 (0)
b760	Control of voluntary movement functions	65 (20)	95 (0)	85 (5)
b765	Involuntary movement functions	15 (25)	50 (0)	55 (10)
b770	Gait pattern functions	70 (20)	100 (0)	90 (0)
b780	Sensations related to muscles and movement functions	75 (20)	100 (0)	95 (0)
s740	Structure of pelvic region	55 (20)	95 (0)	75 (0)
s750	Structure of lower extremity	60 (20)	95 (0)	95 (0)
s770	Additional musculoskeletal structures related to movement	65 (25)	95 (0)	90 (0)
s799	Structures related to movement, unspecified	65 (20)	95 (0)	90 (0)
Activities and participation				
d430	Lifting and carrying objects	70 (15)	85 (10)	75 (5)
d440	Fine hand use	25 (30)	50 (30)	30 (25)
d445	Hand and arm use	30 (25)	50 (25)	30 (20)
d450	Walking	75 (20)	100 (0)	100 (0)
d455	Moving around	70 (30)	95 (0)	80 (0)
d470	Using transportation	75 (15)	85 (0)	80 (5)
d475	Driving	65 (10)	75 (5)	50 (10)
d510	Washing oneself	60 (15)	95 (5)	75 (0)
d530	Toileting	70 (25)	85 (5)	85 (0)
d540	Dressing	65 (20)	80 (10)	75 (0)
d620	Acquisition of goods and services	40 (20)	75 (10)	30 (5)
d640	Doing housework	60 (5)	70 (5)	60 (0)
d660	Assisting others	40 (10)	70 (10)	35 (0)
d770	Intimate relationships	30 (20)	70 (10)	40 (5)
d850	Remunerative employment	70 (20)	90 (0)	95 (0)
d910	Community life	55 (20)	85 (5)	70 (0)
d920	Recreation and leisure	65 (10)	90 (5)	85 (0)
Environmental factors				
e120	Products and technology for personal indoor and outdoor mobility and transportation	75 (10)	80 (0)	95 (0)
e135	Products and technology for employment	55 (10)	65 (0)	70 (0)
e150	Design, construction and building products and technology of buildings for public use	45 (10)	70 (10)	80 (0)
e155	Design, construction and building products and technology of buildings for private use	45 (0)	65 (5)	65 (0)
e225	Climate	20 (25)	30 (15)	25 (20)
e310	Immediate family	45 (25)	55 (5)	30 (5)
e320	Friends	40 (25)	65 (10)	35 (0)
e450	Individual attitudes of health professionals	45 (25)	55 (15)	40 (15)
e460	Societal attitudes	60 (20)	65 (10)	65 (5)
e540	Transportation services, systems and policies	50 (10)	75 (5)	70 (0)
e575	General social support services, systems and policies	60 (5)	80 (5)	70 (0)
e580	Health services, systems and policies	75 (5)	90 (0)	80 (0)
Personal factors				
	Age	80 (5)	90 (0)	100 (0)
	Sex	75 (10)	80 (0)	95 (0)
